# 82 Juvenile dermatomyositis with early-onset anti-MDA 5 antibodies: a case report

**DOI:** 10.1093/rheumatology/keac496.078

**Published:** 2022-10-07

**Authors:** N Ayad, M Gherbi, O Gacem, H Falek, L Labboun, L Amirat, M Chouli, K Abba, H Benmakhlouf, M Achir, M. S Ladj

**Affiliations:** Department of Pediatrics, Djillali Belkhenchir Birtraria Hospital, Algiers—, Algeria

**Keywords:** anti -MDA 5 antibody, juvenile dermatomyositis, interstitial lung disease

## Abstract

**Background:**

Juvenile dermatomyositis (JDM) is a very rare inflammatory myopathy with an approximate incidence of 2–4 per million. The discovery of specific circulating autoantibodies present in about 60% of cases has allowed them to be classified according to their clinical and immunological expressions and their therapeutic response. Anti-Melanoma differentiation associated gene5 (MDA5) antibodies seem to be correlated with a particular clinical phenotype associating cutaneous, respiratory, and articular involvement and most often sparing the muscles.

**Observation:**

We report the case of an early-onset anti-MDA 5 JDM in a 27-month-old girl. The first symptoms appeared at the age of 18 months and consisted of a skin rash and muscle weakness complicated 6 months later by generalized oedema. The child received symptomatic treatments without improvement. At 27 months of age, the child worsened and was admitted for respiratory distress. On clinical examination, she presented with fever and general deterioration, respiratory impairment with swallowing problems, skin damage with specific ulcerations, very significant generalized oedema, and severe muscle damage with axial hypotonia and myogenic EMG tracing. The inflammatory workup was positive and the autoimmune workup revealed 1/320 speckled antinuclear antibody (ANA) and positive anti-MDA5 anti bodies. Chest CT scan showed right interstitial lung disease. The diagnosis of anti-MDA5 JDM was retained and the child was treated with corticosteroids pulses associated with immunoglobulins and methotrexate (MTX) leading to a clear improvement. Corticosteroids and MTX were maintained on a long-term basis.

**Discussion:**

The prevalence of anti-MDA5 anti bodies is variable and depends on ethnic origin (7% in an English cohort *vs* 38% in the Japanese cohort). These antibodies seem to be specific to a particular phenotype of DM associating skin involvement, interstitial lung disease with little or no muscle involvement. The originality of this observation lies in the early age of onset of JDM and in the fact that it expresses the anti-MDA5 antibodies. The skin and muscle involvement was prominent in our patient, while the pulmonary involvement, although not rapidly progressive, justified the combination of bolus methylprednisolone, MTX and immunoglobulins. Despite the delay in diagnosis, we noted a good therapeutic response and stabilization of the disease.

**Conclusion:**

Anti-MDA 5 antibodies must be identified as soon as JDM is diagnosed; their presence is a risk factor for severe lung damage, which must be systematically investigated and treated to improve the prognosis.

